# Oncogenic cancer/testis antigens: prime candidates for immunotherapy

**DOI:** 10.18632/oncotarget.4694

**Published:** 2015-06-30

**Authors:** Morten F. Gjerstorff, Mads H. Andersen, Henrik J. Ditzel

**Affiliations:** ^1^ Department of Cancer and Inflammation Research, University of Southern Denmark, Odense, Denmark; ^2^ Department of Haematology, Center for Cancer Immune Therapy (CCIT), Copenhagen University Hospital, Herlev, Denmark; ^3^ Department of Oncology, Odense University Hospital, Odense, Denmark

**Keywords:** cancer/testis antigen, oncogenesis, immunotherapy

## Abstract

Recent developments have set the stage for immunotherapy as a supplement to conventional cancer treatment. Consequently, a significant effort is required to further improve efficacy and specificity, particularly the identification of optimal therapeutic targets for clinical testing. Cancer/testis antigens are immunogenic, highly cancer-specific, and frequently expressed in various types of cancer, which make them promising candidate targets for cancer immunotherapy, including cancer vaccination and adoptive T-cell transfer with chimeric T-cell receptors. Our current understanding of tumor immunology and immune escape suggests that targeting oncogenic antigens may be beneficial, meaning that identification of cancer/testis antigens with oncogenic properties is of high priority. Recent work from our lab and others provide evidence that many cancer/testis antigens, in fact, have oncogenic functions, including support of growth, survival and metastasis. This novel insight into the function of cancer/testis antigens has the potential to deliver more effective cancer vaccines. Moreover, immune targeting of oncogenic cancer/testis antigens in combination with conventional cytotoxic therapies or novel immunotherapies such as checkpoint blockade or adoptive transfer, represents a highly synergistic approach with the potential to improve patient survival.

## INTRODUCTION

It is clear that most cancers are immunogenic, and that the immune system can, under certain conditions, control tumor growth. However, it appears that in patients with evident disease, the spontaneous immune responses are unable to control tumor growth and, consequently, a significant research effort has been initiated to boost anti-tumor immune responses by therapeutic interventions, e.g. antibodies targeting inhibitory T-cell pathways, cancer vaccines or the adoptive transfer of *in vitro* expanded T cells. Recently, recombinant or chimeric T-cell antigen receptors have been introduced for adoptive T-cell therapy, an approach wherein polyclonal T cells are redirected toward cancer cells that express defined antigens by the transfer of genes encoding those antigen-specific receptors.

T cells are the major effector cells involved in the immune surveillance of cancer by virtue of their ability to detect quantitative and qualitative differences of presented antigens on transformed cells. Indeed, carcinogenic alterations result in an altered protein repertoire. Among the different types of tumor antigens, cancer/testis (CT) antigens represent highly promising therapeutic targets due to a unique set of features. In healthy adults, CT antigen expression is limited to male germ cells, but ectopic expression can be observed in tumor cells of multiple types of human cancer [1-7]. Male germ cells are devoid of HLA-class I molecules and cannot present antigens to T cells [8]. Therefore, CT antigens can be considered neoantigens when expressed in cancer cells and have the capacity to elicit immune responses that are strictly cancer-specific. While the immune-privileged nature of testis germ cells will likely result in decrease or absence of peripheral immune tolerance to CT antigens [9], CT antigen expression has been demonstrated in the medullary thymic epithelial cells that mediate negative selection of self-reactive T cells to tissue-specific proteins [10]. Nonetheless, cellular and humoral immune responses to CT antigens are frequently observed in cancer patients [11-18], and there is an association between CT antigen expression and cytolytic activity of tumor immune infiltrates [19]. Thus, CT antigens represent the promise of highly specific immune targeting of a wide range of human cancers.

In a milestone article (as well as a sequel article a decade later), Hanahan and Weinberg described ‘the hallmarks of cancer’, i.e., the characteristic features of cancer cells, including the capacity for uncontrolled growth (abnormal cell cycle regulation), resistance to death (apoptosis resistance), the potential to migrate and grow at distant sites (metastasis), and the ability to induce the growth of new blood vessels (attract endothelial cells), etc [20, 21]. It is well established that molecular events (genetic and epigenetic changes) are responsible for these cancer traits. In Hanahan and Weinberg's recent update, immune evasion by cancer cells was also included as a hallmark. Our current understanding of tumor immunology suggests that tumor antigen-negative variants may evolve as the result of immunologic pressure, and thus it would be beneficial to target antigens, which, if lost, would reduce the ability of the cancer cells to thrive [22]. Thus, the proteins or protein patterns associated with, or responsible for, these hallmarks of cancer represent ideal targets for therapeutic intervention, including immunotherapy. Several T-cell antigens involved with these cancer traits have been characterized, as exemplified by Cyp1B1, telomerase, survivin (cell division), Bcl-2, Bcl-X(L), survivin, (resistance to apoptosis), RhoC (metastatic potential), survivin, VEGFR (angiogenesis). However, none of them exhibit the cancer-specific expression of CT antigens. Thus, the identification of oncogenic CT antigens (onco-CTAs) is of high priority, but requires in-depth characterization of the molecular and cellular functions of CT antigens. Only recently have critical insights into the function CT antigens in tumor cells emerged that, indeed, suggest the oncogenic functions of multiple CT antigens.

In this article, we review the current understanding of CT antigen functions in oncogenesis and discuss how this novel insight may result in more effective cancer vaccines and new therapeutic strategies.

## THE CANCER/TESTIS ANTIGEN GROUP

MAGE-1 (melanoma-associated antigen 1) was the first CT antigen to be discovered twenty years ago using autologous typing with T-cell clones from a melanoma patient with a favorable clinical course [23]. Further typing of cancer patient T cells and serological analysis of cDNA libraries (SEREX) subsequently lead to the identification of many additional immunogenic CT antigens [24-28]. In recent years, however, additional CT antigens have been added purely based on expression profiling in normal and malignant tissues, and therefore the antigenic properties of many of these remain elusive [29-32]. At present, the known CT antigen group consists of more than 200 proteins [33], a number that is continuously increasing (an overview is provided in Supplementary Table 1 and 2, data from the CTDatabase at http://www.cta.Incc.br).

CT antigens can be categorized into two subclasses depending on the chromosomal localization of their encoding genes. One subclass is the chromosome X-encoded CT antigens (Supplementary Table 1), which are generally highly germ cell-specific and immunogenic. Remarkably, CT antigens constitute more than 10% of the coding sequence on the X chromosome [34], and there appears to have been a strong diversifying selection of CT antigen genes during primate evolution resulting in the generation of CT antigen families with multiple members (MAGE, GAGE, SSX, CT45 etc.) [34, 35]. For instance, the MAGE CT antigens have evolved on three different clusters on chromosome X encoding the MAGE-A, -B and -C subfamilies with 12, 6 and 2 members, respectively [36]. All MAGE proteins contain the MAGE-homology domain, but otherwise exhibit significant differences in structure and function. In contrast to MAGE genes, the GAGE gene cluster is very heterogeneous, comprising from 13 to 39 gene copies encoding highly identical, and likely functionally similar, GAGE proteins [37-39]. Chromosome X-encoded CT antigens tend to be expressed during the fetal stages of germ cell development and in undifferentiated adult male germ cells (i.e. spermatogonia).

The additional subclass of CT antigens is composed primarily of proteins encoded by single-copy genes located on the autosome (BAGE, HAGE, SP17 etc.; Supplementary Table 2). These CT antigens tend to be expressed in meiotic and post-meiotic stages of male germ cells and their expression frequency in cancer is generally lower [40].

Notably, not all CT antigens are strictly limited to male germ cells in normal tissues. Some can be further classified as testis-restricted (only testis), testis-selective (testis and no more than 2 additional tissues) or testis/brain-restricted (expressed in testis and the central nervous system) [3]. How the expression of some CT antigens in a limited number of normal tissues other than testis affects their antigenic properties and potential as therapeutic targets remains largely unresolved.

## ONCOGENIC FUNCTIONS OF CANCER/TESTIS ANTIGENS

**Figure 1:**
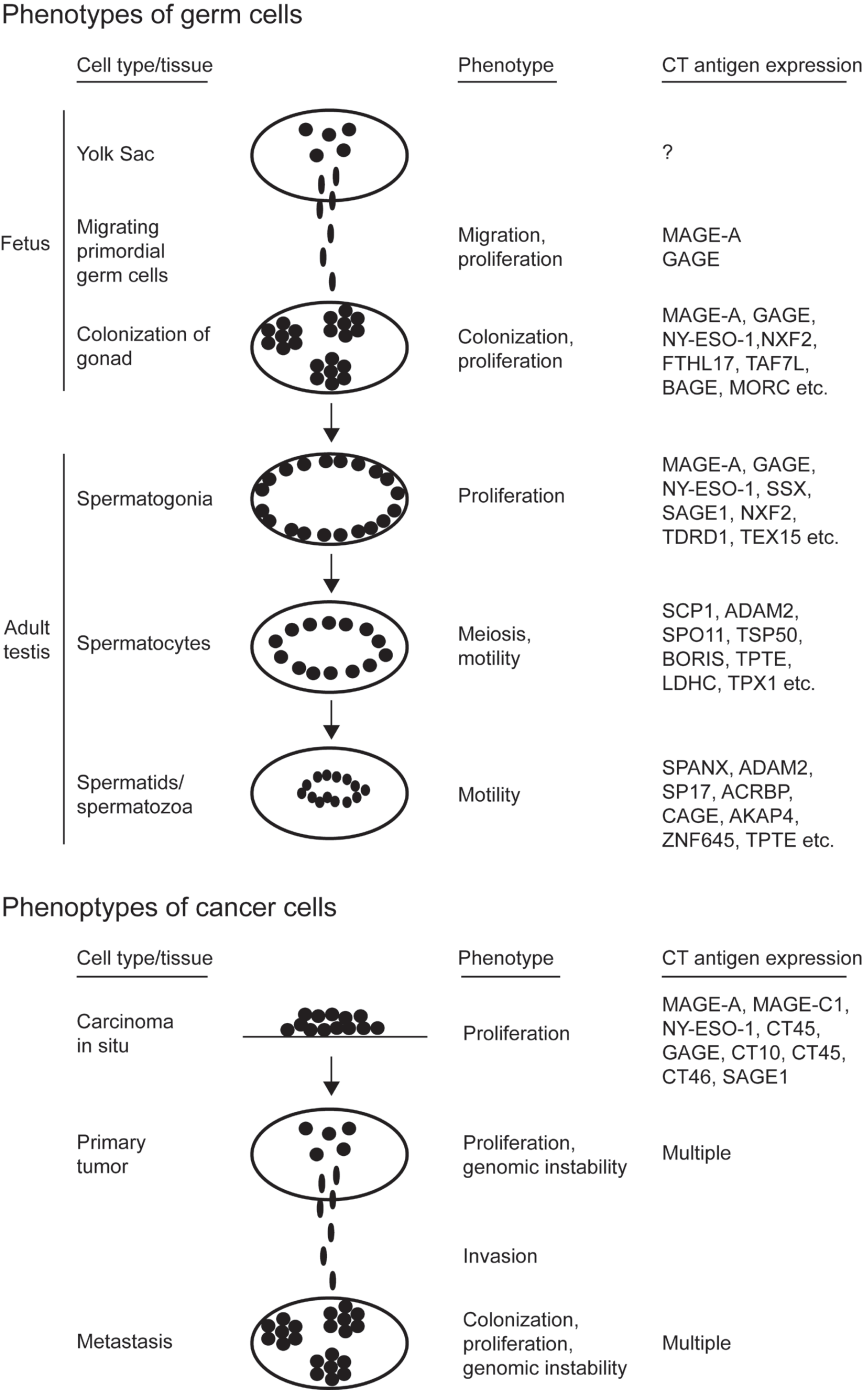
Shared characteristics between germ cells and cancer cells Germ cells and cancer cells share many features that are absent in most other cell types, suggesting that germ cell programs contribute to cancer development and progression. In addition to cancer/testis antigen expression, these characteristics include proliferation, migration, colonization and meiosis/genomic instability.

**Figure 2:**
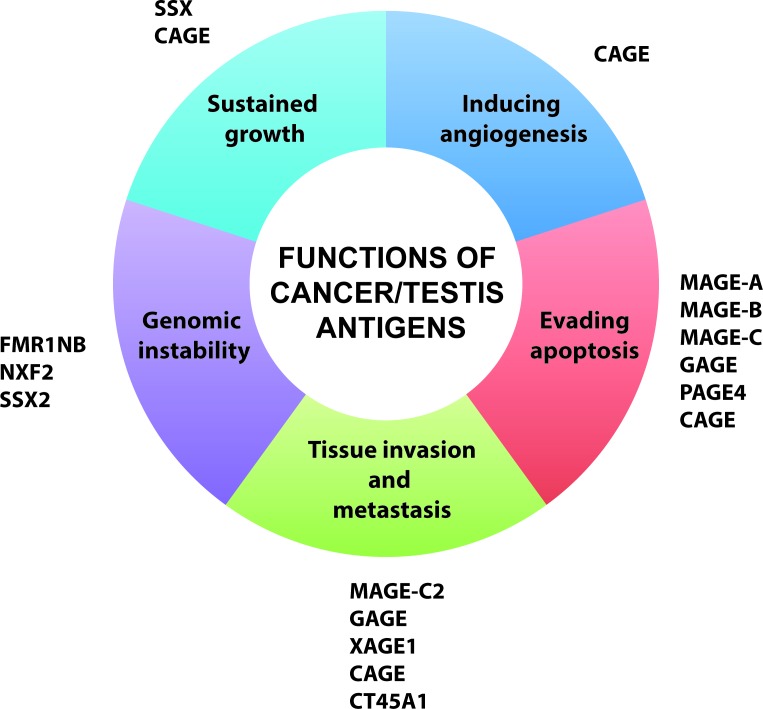
Oncogenic functions of cancer/testis antigens Tumorigenesis involves acquisition of a specific set of essential capabilities (as described in the ‘Hallmarks of cancer’ by Hanahan and Weinberg”[20, 21]). These include uncontrolled growth, resistance to death (apoptosis), the potential to migrate and grow at distant sites (invasion and metastasis), the ability to induce the growth of new blood vessels (induce angiogenesis), etc. Underlying these hallmarks is genomic instability, which generates the genetic variation that accelerates their acquisition. Cancer/testis antigens confer several of these important capabilities to cancer cells, suggesting that they are directly implicated in tumorigenesis.

Cells of the germ line and the closely related trophoblasts share many features with cancer cells (Figure 1). For instance, when primitive germ cells (i.e. primordial germ cells) arise in the wall of the yolk sac they are highly motile and able to penetrate tissues as they journey to the gonadal primordium [41]. This process of germ cell colonization of the gonad in many ways resembles the progression of cancer cells from primary tumor to metastasis. Moreover, during spermatogenesis, germ cells exhibit characteristics similar to cancer cells. The immature spermatogonia maintain their proliferative capacity throughout life and continuously differentiate into spermatocytes, which go through meiosis comparable to the chromosomal changes observed in most cancers. Trophoblasts also exhibit overlapping features with cancer cells, as they are invasive and burrow into the endometrium to implant the embryo, as well as proliferate vigorously to form the non-maternal part of the placenta. These observations led to the hypothesis that activation of embryonic or gametogenic programs are among the driving forces of tumorigenesis [42], a concept supported by the fact that many germ cell and placental proteins, including CT antigens, are aberrantly expressed in cancer [43, 44]. In recent years, the functions of CT antigens have received extensive investigation, and the emerging data are consistent with the concept that reactivation of germline genes might confer central characteristics to cancer cells (Figure 2).

### Multiple cancer/testis antigens affect cancer cell growth

Sustained growth is an important feature of all cancer cells and the net result of deregulation of the delicate cell cycle machinery that balances proliferative signaling and cell survival signals. Importantly, multiple CT antigens have been shown to support cancer cell growth. We have recently shown that knockdown of the CT antigen SSX2 in melanoma cancer cells significantly reduces cellular proliferation [45]. This finding was supported by another study demonstrating that SSX proteins activate several important growth pathways, such as MAPK and Wnt [46]. SSX2 is a DNA binding protein that associates with and regulates the structure of chromatin [47]. This includes, but is likely not limited to, antagonistic regulation of the function of Polycomb Group proteins, which are important epigenetic regulators of gene expression and implicated in cancer development [48]. Thus, current results suggest that SSX proteins support cancer cell proliferation through regulation of gene expression. The CAGE CT antigen also seems to promote cell proliferation as it has been shown to enhance Cyclin D1 and E levels, thereby stimulating cell cycle progression [49]. Furthermore, CAGE have angiogenic potential, which may indirectly support cancer cell growth [50].

MAGE proteins are among the functionally and immunologically most well-characterized CT antigens, and several members of the MAGE CT antigen family have been implicated in cancer cell survival [51-60]. Most importantly, MAGE CT antigens bind and regulate the function of the essential tumor suppressor p53, thereby increasing survival of cancer cells. Knockdown of MAGE-A, -B and -C proteins in melanoma cells inhibited complexing of p53 and its co-repressor KAP1, leading to increased p53 activity and apoptosis [59]. Further, MAGE-A proteins have been demonstrated to directly block the interaction of p53 with chromatin, thereby inhibiting its effect as a transcriptional regulator [60], and to strongly down-regulate p53 function by recruiting transcription repressors [histone deacetylases (HDACs)] to p53-regulated genes [57]. In addition, multiple MAGE family proteins have been identified as functional regulators of E3 RING ubiquitin ligases, which facilitate the proteasomal degradation of many proteins, including p53 [58]. Members of the GAGE-like CT antigen family (GAGE and PAGE proteins) also confer apoptotic resistance to various types of cancer cells. GAGE7 prevented apoptosis in response to different types of apoptotic stimuli [55, 61, 62], and knockdown of PAGE4 induced cell death and attenuated tumor growth *in vivo* [63].

Cellular senescence is a state of irreversible growth arrest that can occur, for example, in response to excessive oncogene stimulation, and seems to be an important tumor suppressor mechanism [64]. Currently, little is known about how cells bypass the senescence response in tumorigenesis, although a growing body of evidence implicates the involvement of Mage-A2. In human fibroblasts, Mage-A2 was found to maintain cell proliferation in response to RasV12 expression by limiting the senescence response to this oncogene [65]. This effect of Mage-A2 was also attributed to its inhibition of p53 function, suggesting that MAGE-A2 could play a novel role in early progression to malignancy by interfering with p53 function. This may block the senescence program, a critical barrier against cell transformation. Further studies addressing the potential cooperation between CT antigens interfering with cell cycle regulation and oncogenic signaling should be of high priority.

The above results show that several CT antigens may support tumor growth, but also indicate that CT antigens may be important in treatment responses to cytotoxic or growth inhibitory anti-cancer drugs. Clearly, all CT antigens that enhance cell survival may decrease the effectiveness of treatment with cytotoxic agents. This has been demonstrated with MAGE-A, MAGE-C, GAGE, PAGE-4 and CAGE, which render cells resistant to DNA damage-inducing drugs commonly used in the clinic, such as etoposide and paclitaxel [52, 55, 57, 61, 63, 66], as well as additional cytotoxic drugs used in cancer treatment [51, 52, 61]. MAGE-A proteins have also been proposed to be involved in development of tamoxifen-resistance in breast cancer since they are up-regulated in tamoxifen-resistant clones and knockdown of MAGE-A2 sensitized cells to tamoxifen. Further, significant association between MAGE-A expression and reduced overall survival in a series of estrogen receptor-positive, tamoxifen-treated, breast cancer patients was observed [67]. Thus CT antigens may be considered as both prognostic and predictive markers.

### Cancer/testis antigens and metastasis formation

A clear link between CT antigen expression and cancer progression has been observed [7, 68-71]. CT antigens are rarely expressed in benign neoplastic lesions, such as carcinoma *in situ* and melanocytic nevi [72], but are frequently expressed in primary melanoma, and even more frequently in metastases. For example, the frequency of MAGE-A1 and -A4 expression in primary melanoma tumors were 20% and 9%, respectively, but 51% and 44%, respectively, in distant metastases [71]. This indicates that CT antigens may play a direct role in the highly complex process of metastasis, which involves several steps, including local invasion, intravasation into blood and lymphatic vessels, survival in the circulation, extravasation into distant tissues and colonization. An important feature of cells with metastatic capability is increased motility and invasive potential. Interestingly, several CT antigens, including MAGE-C2, GAGE, XAGE1, CAGE and CT45A1, have been demonstrated to enhance both phenotypes [73-75]. Epithelial-to-mesenchymal transition (EMT) is a process by which epithelial cells lose their cell-cell adhesion and cell polarity, and gain migratory and invasive properties characteristic of mesenchymal cells. EMT has been shown to be instrumental for metastatic progression of several types of cancer, especially melanoma [76]. Molecular analysis of the effects of MAGE-C2 expression in breast cancer cells revealed signs of EMT (i.e. reduced E-cadherin, reduced cytokeratin, increased vimentin and increased fibronectin) [73]. Similarly, SSX, CAGE and CT45A1 have been shown to regulate the function of central EMT proteins (i.e. beta-catenin, SNAIL and TWIST) [46, 74, 77], and may support the transition to a metastatic phenotype. In contrast to melanocyte differentiation antigens (i.e. MART and GP100), which are down-regulated during EMT, CT antigens have been shown to be up-regulated or unchanged in expression [78]. This is consistent with the enhanced expression of CT antigens in metastases relative to primary tumors, and suggests that CT antigens may be prime candidates for blocking metastatic progression or targeting cancer cells in established metastatic lesions.

GAGE proteins are the only CT antigens that have been identified in both migrating primordial germ cells and trophoectodermal cells [79, 80], which are highly motile and invasive. Furthermore, knockdown of GAGE proteins in melanoma cells lines greatly reduce their migration and invasion. We, and others, have found that GAGE proteins are highly up-regulated in metastatic clones of isogenic breast and gastric cancer models, further suggesting a role in metastasis formation [81]. However, direct involvement of GAGE proteins in metastasis remains to be demonstrated. Further elucidating the effect of GAGE proteins as well as additional CT antigens on metastases formation in *in vivo* models should be of high priority.

### Cancer/testis antigens and genomic integrity

The genome of cancer cells is unstable and constantly subject to mutations, chromosomal rearrangements and loss or gain of chromosomes. This genomic instability is largely responsible for the generation of mutant genotypes that confer selective advantages on cell subclones that, in turn, supports tumorigenesis. The extent to which different mechanisms affect genomic instability is controversial, but DNA double-strand breaks and abnormal segregation of chromatids during mitosis are generally accepted to be involved. In addition, the meiotic process involves the generation of double-strand breaks during the exchange of genetic material between sister chromatids and pairing and segregation of chromatids. Therefore, it can be speculated that activation of meiotic programs in cancer cells may contribute to genome instability, and that meiosis-specific CT antigens, such as SPO11, SCP1 and HORMAD1, may be involved [31, 82, 83]. SPO11 is instrumental to meiotic chromatid exchange of genetic material by producing double strand breaks [84] and might promote chromosomal rearrangements in cancer cells by a similar mechanism. SCP1 and HORMAD1 are involved in chromosome pairing in the context of meiosis, and their presence in somatic cells may deregulate mitosis. Interesting, meiotic proteins have been implicated in reducing polyploidy in cancer cells and may serve as a means to maintain the balance between increased genome instability driving genetic variation and decreased genome instability necessary for propagating these malignant clones [85-87].

A number of additional CT antigens as well as other proteins preferentially expressed in testis have been shown to support productive mitosis in cancer cells. For instance, FMR1NB-, NXF2- STARD6- and FSIP1-depletion in lung cancer cells enhanced the occurrence of mitotic arrest and micronucleation in response to induction of mitotic stress induced by Paclitaxel or Nocodazole treatment [88, 89]. Thus, selected CT antigens may be essential for mitotic fidelity and resistance to chemotherapeutics in cancer cells. Interestingly, overexpression of NXF2, STARD6 and FSIP1 also induced mitotic defects, and we have recently shown that the overexpression of the SSX2 CT antigen is associated with mitotic aberrations and genomic instability [45]. These results suggest that multiple CT antigens and testis proteins support delicate processes in cancer cells that, when disturbed, lead to aberrant mitotic phenotypes [88]. Such CT antigens may represent novel targets for anti-cancer therapy.

## ONCOGENIC CANCER/TESTIS ANTIGENS AS TARGETS FOR IMMUNOTHERAPY

### Cancer/testis antigen vaccination

**Figure 3:**
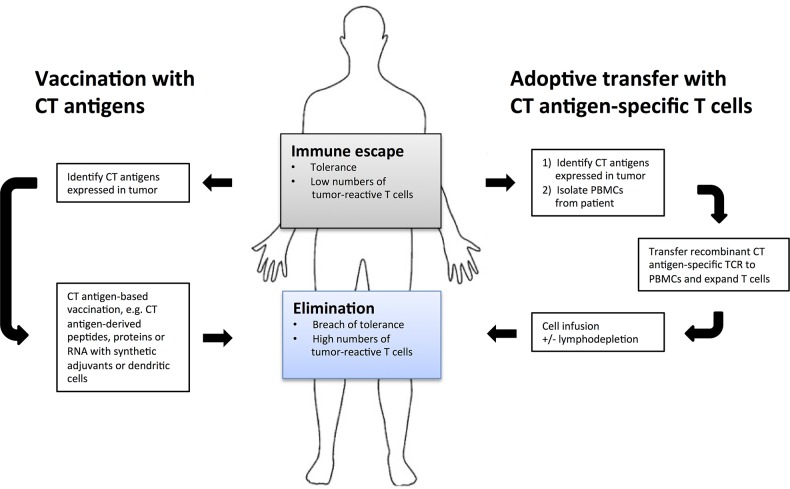
Cancer immunotherapies targeting cancer/testis antigens The goal of therapeutic cancer immunotherapy is to reverse immune escape of tumor cells by overcoming tolerance and increasing numbers of tumor-reactive T cells. This can be accomplished by boosting existing immune responses to tumor antigens, and cancer/testis antigens have proved useful as targets due to high tumor-specificity and immunogenicity. Currently two types of cancer/testis antigen-specific immunotherapy are used: vaccination and adoptive transfer. Vaccination stimulates the patient's intrinsic immune response to cancer/testis antigens expressed by their tumor by administration of immunogenic peptides/proteins loaded on dendritic cells or in combination with adjuvants. Cancer/testis antigens are also popular targets for adoptive transfer wherein recombinant T-cell receptors specific for cancer/testis antigen epitopes are inserted into patient T cells, which are then expanded and transferred back to patients. Targeting oncogenic cancer/testis antigens in vaccination and adoptive transfer regimens may greatly reduce the risk of outgrowth of escape variants.

The immunogenicity and cancer-specificity of CT antigens have made them prioritized targets for cancer immunotherapy, and their therapeutic function has been tested in a variety of clinical settings (reviewed in Gjerstorff et al. [90]). For historical reasons, MAGE-A and NY-ESO-1 in particular have been tested extensively as components in therapeutic cancer vaccines (Figure 3). CT antigen vaccines are generally well-tolerated, and immunological responses as well as some clinical responses have been obtained. There are presently a large number of ongoing cancer vaccination trials assessing the therapeutic efficacy of CT antigens. For instance, 35 open clinical studies on NY-ESO-1 are listed at http://www.clinicaltrials.gov. Some of the investigated CT antigens have known oncogenic effects (e.g. MAGE-A, MAGE-C2, SSX2, PRAME), whereas the functions in cancer of some CT antigens such as NY-ESO-1 remain unknown.

Several recent clinical trials have provided encouraging results. Treatment of advanced melanoma and ovarian cancer patients with a recombinant fowlpox-NY-ESO-1 vaccine resulted in NY-ESO-1 antibody and CD4+ and CD8+ T-cell responses in the majority of these patients, and CD8+ responses correlated with progression-free survival [91, 92]. Another study tested the interesting approach of delivering NY-ESO-1 to dendritic cells by fusing the antigen to an antibody specific for the dendritic cell surface molecule, CD205, in combination with Toll-like receptor agonists. Again, humoral and T-cell responses were obtained in patients with mixed advanced cancers, and 13 of 42 patients experienced disease stabilization [93]. This study also suggested that this treatment enhanced the effect of subsequent treatment with CTLA-4 immune checkpoint inhibitors.

The most comprehensive investigation used MAGE-A3 as a vaccine target in a randomized, double-blinded, placebo-controlled phase III study in 2,272 non-small cell lung cancer (NSCLC) patients (MAGRIT). Recombinant MAGE-A3-influenzae protein D fusion protein and the immunostimulant AS15 were administered to patients in up to 13 intramuscular injections over a period of 27 months. Unfortunately, this trial was terminated in March 2014 (ESMO Congress 2014, Abstract 1173O), since it was shown that the vaccine did not significantly extend disease-free survival (DFS) compared to placebo. The same vaccine was also tested in another phase III clinical trial in 1,345 MAGE-A3-positive melanoma patients (DERMA), and this likewise failed with no improvement in DFS in the overall population (http://www.gsk.com). Why these two clinical studies were unsuccessful remains to be clearly determined, but the failure to induce sufficient cell-mediated responses may result from either the generally low immunogenicity of MAGE-A3 or the inability of the chosen vaccine design to break immune tolerance. The lack of effect may indeed advocate the need for more careful selection of tumor antigen targets. MAGE-A3 has been the most widely studied CT-antigen, but has only been shown to exhibit low immunogenicity. In addition, although MAGE-A3 has been demonstrated to possess tumor-promoting capabilities, functional studies have only been performed with thyroid and pituitary cancer cells [94, 95] and, to our knowledge, functional studies on MAGE-A3 have not been performed using NSCLC or melanoma cells.

Thus far, the majority of anti-cancer vaccination trials have targeted only a single antigen. However, recent data suggests that the clinical impact of immunotherapy increases by the inclusion of several antigens [96]. Thus, an exciting strategy would be to co-target biologically-connected proteins, e.g. several CT antigens involved in cancer cell survival, in a multi-epitope setting. This may increase the magnitude and flexibility of the vaccine-induced anti-tumor response and prevent the escape of tumor cells that would otherwise occur through selective loss of single target antigens. Although multiple CT antigens are up-regulated in many cancers, the amount of each antigen differs within individual cancer cells in the same tumor and between tumors of different patients with the same cancer type. Therefore, simultaneous targeting of these proteins may be a more effective strategy than targeting either molecule alone. It is not presently known how the redundancy of the many MAGE-A proteins or additional co-expressed CT antigens in regulation of survival pathways may affect tumor responsiveness to anti-MAGE-A3 treatment. In that respect, it will be interesting to see the outcome of ongoing trails evaluating the effect of vaccines targeting multiple antigens affecting cancer cell growth and survival. In a completed, but unreported, study, several MAGE family members (i.e. MAGE-A1, MAGE-A3, MAGE-A4, MAGE-A10 and MAGE-C2) were targeted simultaneously in melanoma patients using a peptide vaccine. Additional studies will target different proteins affecting proliferation and survival using autologous T cells (i.e. MAGE-A4, SSX, PRAME and survivin) in lymphoma, myeloma and mixed solid cancer patients (http://www.clinicaltrials.gov identifiers: NCT00365937, NCT01333046, NCT02239861, NCT02291848). Future studies should focus on regimens integrating emerging knowledge about tumor antigen function and immune stimulation to generate optimal anti-tumor responses.

Although individualized cancer vaccines targeting mutation antigens are currently receiving a great deal of attention, there may be several advantages of polyvalent CT antigen vaccines. The use of shared antigens, such as oncogenic CT antigens, as vaccine targets does not necessitate the tedious and time-consuming genome sequencing required to identify patient-specific antigens and antigen synthesis. Polyvalent CT antigen vaccines can be administered early after diagnosis and prevent further progression of the tumor before initiation of treatment. Furthermore, CT antigens may be preferred targets for types of cancer with low prevalence of somatic mutations [97].

### Cancer/testis antigens for adoptive T-cell therapy

Administration of *in vitro* expanded infiltrating T cells from melanoma tumors following myeloablative lymphodepleting regimens have been shown to induce tumor regression in more than 50% of patients with metastatic melanoma, and durable complete responses have also been achieved [98]. A similar approach for other types of human cancer is complicated by the difficulties with obtaining tumor-infiltrating lymphocytes. To expand this approach to a broader patient population, tumor-reactive T cells can be generated by genetically modifying autologous T cells to express recombinant or chimeric T-cell receptors directed against common tumor antigens, a type of therapy for which CT antigens are among the high priority targets (Figure 3). Their frequent expression in many types of cancer makes CT antigen-directed T-cell therapy applicable to many patients, and their restriction in normal tissues to the HLA class I-negative germ cells likely prevents adverse effects. T-cell receptors against epitopes from MAGE-A4, NY-ESO-1 and SSX2 have been developed and are currently under evaluation for clinical function [99-101]. Preliminary results have demonstrated that clinical responses can indeed be achieved with CT antigen-directed T-cell therapy. For instance, patients with NY-ESO-1-positive melanoma and synovial sarcoma receiving autologous T cells transduced with an NY-ESO-1-specific T-cell receptor demonstrated clinical responses in 55% and 61% of the cases, respectively. This strategy did not cause any cytotoxicity in patients. However, other clinical trials with modified autologous T cells have highlighted the importance of target specificity. Although MAGE-A3 exhibits high cancer cell specificity, severe toxicity of MAGE-A3-directed T-cell therapy has been reported in two instances. In one trial, two patients died from severe brain damage apparently due to cross-reactivity of the T-cell receptor-transduced PBMCs with a highly similar epitope from MAGE-A12, which is expressed in tissues of the normal brain [102]. Similarly, two patients experienced cardiac arrest after treatment with autologous PBMCs transduced with a modified MAGE-A3 T-cell receptor, which apparently cross-reacted with an epitope of the cardiac protein titin [103, 104]. Although these unfortunate events can be attributed to the lack of T-cell receptor-specificity, they also emphasize the need for absolute target-specificity, suggesting that CT antigens and mutation antigens may be appropriate targets for this highly potent strategy.

## COMBINATION THERAPIES WITH ONCOGENIC CANCER/TESTIS ANTIGENS

**Figure 4:**
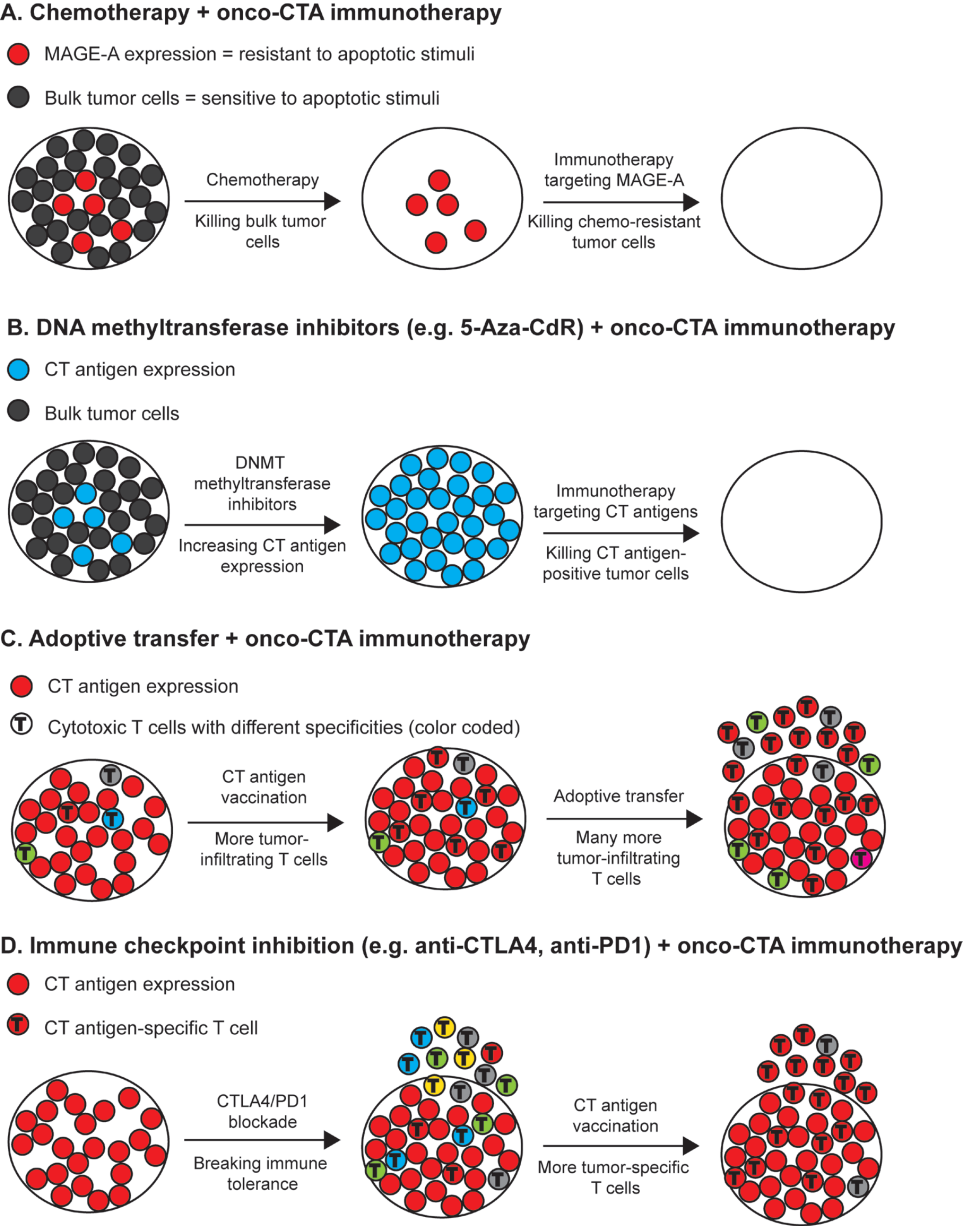
Targeting oncogenic cancer/testis antigen in combination with other therapies **A.** Chemotherapy may only kill the bulk of tumor cells, leaving chemoresistant cells. Since several cancer/testis antigens have been demonstrated to inhibit apoptosis in cancer cells and lower the response to cytotoxic anti-cancer drugs, targeting cancer/testis antigens in combination with chemotherapy may be effective. **B.** DNA methyltranferase (DNMT) inhibitors, which have been approved for treatment of hematologic malignancies, can induce cancer/testis antigen expression in cancer cells and reverse intratumoral heterogeneity. Thus, treatment with DNMT inhibitors may boost the effect of vaccines targeting oncogenic cancer/testis antigens. **C.** The foremost limitation to expanding adoptive transfer of T cells to treatment of multiple types of human cancer is the need for sufficient numbers of tumor-infiltrating T cells. Vaccination with cancer/testis antigens expressed in the tumor can increase these numbers in cancer types wherein such cells are sparse. Thus, the combination of adoptive transfer and cancer/testis antigen vaccination may be beneficial. **D.** Tumor antigen vaccination can enhance the effect of immune-checkpoint blockade, and directing the immune response to antigens important for the tumor cells, such as oncogenic cancer/testis antigens, may further enhance the effect of this combination treatment.

Strategies that combine current knowledge of conventional treatments with data on how these treatments impact the T cell population and the immune system are important elements in the development and optimization of novel treatment regimens, which should also include novel insight into the biology of CT antigens. Chemotherapeutic agents impact not only tumor cell proliferation but also cell survival, and it is evident that various drugs can kill tumor cells by activation of common apoptotic pathways. Essentially all cytotoxic anticancer drugs, e.g. microtubule binding drugs, DNA-damaging agents and nucleosides, induce apoptosis of malignant cells, but drug resistance is a major limiting factor of chemotherapeutics that may also lead to cross-resistance to other drugs with different mechanisms of action [105]. Cancer-associated defects in apoptosis play a vital role in drug resistance [106]. Interestingly, as described above, several CT antigens have been associated with impaired apoptosis [51-61, 63, 66]. Anti-apoptotic CT antigens are prime candidates for immunotherapy since they facilitate the escape of malignant cells from cytotoxic therapies. Thus, the combination of immunotherapy targeting anti-apoptotic CT antigens with conventional chemotherapy appears to be highly synergistic. In an ideal combinational therapeutic setting, conventional therapy would kill the majority of the cancer cells, leaving only cells that express high levels of CT antigens, which would be particularly vulnerable to killing by therapy-induced CT antigen-specific T cells (Figure 4A). The synergy of these measures would, consequently, result in a more effective treatment than either regime alone.

Spontaneous immunity against CT antigens can be introduced as a response to treatment with DNA methyltransferase (DNMT) inhibitors, such as 5-aza-2′-deoxycytidine (5-aza-2′-CdR) or 5-azacytidine [107, 108], which have been approved for treatment of hematologic malignancies and may also be useful for solid cancers. These agents have the ability to reverse epigenetic silencing of genes in cancer cells and restore normal function of multiple cellular processes, including cell-cycle regulation, apoptosis and immune recognition [109, 110]. DNMT inhibitors have been described to up-regulate expression of many CT antigens, e.g. MAGE, GAGE, NY-ESO-1 and PRAME [107], and such up-regulation has been associated with induction of MAGE-specific T-cell responses in acute myeloid leukemia and myeloid dysplastic syndrome patients [107]. Furthermore, PRAME antigen-specific killing of malignant cell lines by low-avidity CTL clones have been shown to be increased following treatment with DNMT inhibitors [111]. The clinical and prognostic significance of these CT-antigen-specific T-cell responses on the clinical efficacy of DNMT inhibitor treatment in hematologic malignancies remains to be defined, although it suggests that combining DNMT inhibitors with vaccination to induce CT antigen expression in both tumors and CT antigen-specific T cells may prove beneficial (Figure 4B). However, as novel insight has demonstrated that some CT antigens can also confer tumorigenic properties to cancer cells, DNMT inhibitors should be used with caution. Although the net result seems to be an inhibitory effect on tumor function, there may be unidentified deleterious effects, such as enhanced EMT, which supports metastasis.

Another utilization of the up-regulation of CT antigens by DNMT inhibitors may be combining DNMT inhibitors with adoptive transfer of CT-specific T cells. Accordingly, the combination of adoptive transfer of MAGE-A4-specific T cells with 5-aza-2′-CdR in patients with Hodgkin's lymphoma generated MAGE-A4-specific T cells and increased their anti-tumor T-cell repertoire [112]. Similarly, combinations of DNMT inhibition and CT antigen vaccination may enhance the frequency of tumor-specific T cells in tumors and thereby support adoptive T-cell transfer regimens (Figure 4C). As described above, genetically modifying autologous T cells to express recombinant T-cell receptors is an additional approach to target CT antigens, and may also naturally function synergistically with DNMT inhibition.

Antibodies that block the function of immune checkpoint molecules (i.e. CTLA-4 and PD1), resulting in T-cell activation, have greatly improved survival in metastatic melanoma as well as NSCLC, and are being tested for treatment of several additional types of cancer [113-115]. This demonstrates that the immune system can be modulated to effectively control tumor growth, although a significant number of patients do not respond to this treatment or experience relapse. Recent results suggest that augmented anti-tumor effects can be obtained by combining immune checkpoint blockade with vaccination [93, 116-119]. Thus, vaccination with CT antigens, and oncogenic CT antigens in particular, should clearly be considered in combination with immune checkpoint inhibition for enhanced effects (Figure 4D).

## CONCLUSIONS

The frequent use of CT antigens in vaccination trials have been based on their high cancer specificity, immunogenicity and relatively frequent expression in many different types of human cancer. As discussed in this review, recent results show that multiple CT antigens exhibit characteristics important for tumorigenesis, suggesting that targeting such oncogenic CT antigens may control cancer development. For instance, the targeting of CT antigens that enhance cancer cell growth and/or survival would diminish the tumorigenic potential. Likewise, targeting of CT antigens that promote EMT may reduce the risk of metastases. Thus, oncogenicity should be considered an important criterion for selection of CT antigens for future immunotherapy, and functional characterization of CT antigens should be of high priority in order to identify more oncogenic CT antigen targets or CT antigens expressed by cancer cell subpopulations important for tumor initiation and progression.

Recent advances in breaking immune tolerance in cancer patients using immune checkpoint inhibitors has set the stage for immune targeting as a supplement to conventional treatment of human cancer, and a significant effort remains to further improve efficacy and specificity of treatment. Overall, the synergistic effects of conventional and immunological therapies necessitate re-thinking current clinical strategies both with respect to the chosen chemotherapeutics and the design of the selected immunotherapy. Thus, targeting oncogenic CT antigens seems to be a promising, broadly applicable approach that can synergistically be combined with conventional cytotoxic therapies or novel types of immunotherapy such as checkpoint blockade or adoptive transfer.

## SUPPLEMENTARY MATERIAL TABLES



## References

[1] Chen YT, Hsu M, Lee P, Shin SJ, Mhawech-Fauceglia P, Odunsi K, Altorki NK, Song CJ, Jin BQ, Simpson AJ, Old LJ (2009). Cancer/testis antigen CT45: analysis of mRNA and protein expression in human cancer. International journal of cancer Journal international du cancer.

[2] dos Santos NR, Torensma R, de Vries TJ, Schreurs MW, de Bruijn DR, Kater-Baats E, Ruiter DJ, Adema GJ, van Muijen GN, van Kessel AG (2000). Heterogeneous expression of the SSX cancer/testis antigens in human melanoma lesions and cell lines. Cancer research.

[3] Hofmann O, Caballero OL, Stevenson BJ, Chen YT, Cohen T, Chua R, Maher CA, Panji S, Schaefer U, Kruger A, Lehvaslaiho M, Carninci P, Hayashizaki Y, Jongeneel CV, Simpson AJ, Old LJ (2008). Genome-wide analysis of cancer/testis gene expression. Proceedings of the National Academy of Sciences of the United States of America.

[4] Sahin U, Tureci O, Chen YT, Seitz G, Villena-Heinsen C, Old LJ, Pfreundschuh M (1998). Expression of multiple cancer/testis (CT) antigens in breast cancer and melanoma: basis for polyvalent CT vaccine strategies. International journal of cancer Journal international du cancer.

[5] Zendman AJ, de Wit NJ, van Kraats AA, Weidle UH, Ruiter DJ, van Muijen GN (2001). Expression profile of genes coding for melanoma differentiation antigens and cancer/testis antigens in metastatic lesions of human cutaneous melanoma. Melanoma research.

[6] Gjerstorff MF, Johansen LE, Nielsen O, Kock K, Ditzel HJ (2006). Restriction of GAGE protein expression to subpopulations of cancer cells is independent of genotype and may limit the use of GAGE proteins as targets for cancer immunotherapy. British journal of cancer.

[7] Greve KB, Pohl M, Olsen KE, Nielsen O, Ditzel HJ, Gjerstorff MF (2014). SSX2-4 expression in early-stage non-small cell lung cancer. Tissue antigens.

[8] Janitz M, Fiszer D, Michalczak-Janitz K, Lukaszyk A, Fernandez N, Skorupski W, Kurpisz M (1994). Analysis of mRNA for class I HLA on human gametogenic cells. Molecular reproduction and development.

[9] Fijak M, Meinhardt A (2006). The testis in immune privilege. Immunological reviews.

[10] Gotter J, Brors B, Hergenhahn M, Kyewski B (2004). Medullary epithelial cells of the human thymus express a highly diverse selection of tissue-specific genes colocalized in chromosomal clusters. The Journal of experimental medicine.

[11] Tsuji T, Altorki NK, Ritter G, Old LJ, Gnjatic S (2009). Characterization of preexisting MAGE-A3-specific CD4+ T cells in cancer patients and healthy individuals and their activation by protein vaccination. Journal of immunology.

[12] Andersen RS, Thrue CA, Junker N, Lyngaa R, Donia M, Ellebaek E, Svane IM, Schumacher TN, Thor Straten P, Hadrup SR (2012). Dissection of T-cell antigen specificity in human melanoma. Cancer research.

[13] Wang Y, Wu XJ, Zhao AL, Yuan YH, Chen YT, Jungbluth AA, Gnjatic S, Santiago D, Ritter G, Chen WF, Old LJ, Ji JF (2004). Cancer/testis antigen expression and autologous humoral immunity to NY-ESO-1 in gastric cancer. Cancer immunity.

[14] Akcakanat A, Kanda T, Koyama Y, Watanabe M, Kimura E, Yoshida Y, Komukai S, Nakagawa S, Odani S, Fujii H, Hatakeyama K (2004). NY-ESO-1 expression and its serum immunoreactivity in esophageal cancer. Cancer chemotherapy and pharmacology.

[15] Ayyoub M, Rimoldi D, Guillaume P, Romero P, Cerottini JC, Valmori D, Speiser D (2003). Tumor-reactive, SSX-2-specific CD8+ T cells are selectively expanded during immune responses to antigen-expressing tumors in melanoma patients. Cancer research.

[16] Gnjatic S, Atanackovic D, Jager E, Matsuo M, Selvakumar A, Altorki NK, Maki RG, Dupont B, Ritter G, Chen YT, Knuth A, Old LJ (2003). Survey of naturally occurring CD4+ T cell responses against NY-ESO-1 in cancer patients: correlation with antibody responses. Proceedings of the National Academy of Sciences of the United States of America.

[17] Qian F, Gnjatic S, Jager E, Santiago D, Jungbluth A, Grande C, Schneider S, Keitz B, Driscoll D, Ritter G, Lele S, Sood A, Old LJ, Odunsi K (2004). Th1/Th2 CD4+ T cell responses against NY-ESO-1 in HLA-DPB1*0401/0402 patients with epithelial ovarian cancer. Cancer immunity.

[18] Milne K, Barnes RO, Girardin A, Mawer MA, Nesslinger NJ, Ng A, Nielsen JS, Sahota R, Tran E, Webb JR, Wong MQ, Wick DA, Wray A, McMurtrie E, Kobel M, Kalloger SE (2008). Tumor-infiltrating T cells correlate with NY-ESO-1-specific autoantibodies in ovarian cancer. PloS one.

[19] Rooney MS, Shukla SA, Wu CJ, Getz G, Hacohen N (2015). Molecular and genetic properties of tumors associated with local immune cytolytic activity. Cell.

[20] Hanahan D, Weinberg RA (2000). The hallmarks of cancer. Cell.

[21] Hanahan D, Weinberg RA (2011). Hallmarks of cancer: the next generation. Cell.

[22] Schreiber RD, Old LJ, Smyth MJ (2011). Cancer immunoediting: integrating immunity's roles in cancer suppression and promotion. Science.

[23] van der Bruggen P, Traversari C, Chomez P, Lurquin C, De Plaen E, Van den Eynde B, Knuth A, Boon T (1991). A gene encoding an antigen recognized by cytolytic T lymphocytes on a human melanoma. Science.

[24] Park S, Lim Y, Lee D, Cho B, Bang YJ, Sung S, Kim HY, Kim DK, Lee YS, Song Y, Jeoung DI (2003). Identification and characterization of a novel cancer/testis antigen gene CAGE-1. Biochimica et biophysica acta.

[25] Chen YT, Gure AO, Tsang S, Stockert E, Jager E, Knuth A, Old LJ (1998). Identification of multiple cancer/testis antigens by allogeneic antibody screening of a melanoma cell line library. Proceedings of the National Academy of Sciences of the United States of America.

[26] Jager D, Unkelbach M, Frei C, Bert F, Scanlan MJ, Jager E, Old LJ, Chen YT, Knuth A (2002). Identification of tumor-restricted antigens NY-BR-1, SCP-1, and a new cancer/testis-like antigen NW-BR-3 by serological screening of a testicular library with breast cancer serum. Cancer immunity.

[27] De Backer O, Arden KC, Boretti M, Vantomme V, De Smet C, Czekay S, Viars CS, De Plaen E, Brasseur F, Chomez P, Van den Eynde B, Boon T, van der Bruggen P (1999). Characterization of the GAGE genes that are expressed in various human cancers and in normal testis. Cancer research.

[28] Chen YT, Scanlan MJ, Sahin U, Tureci O, Gure AO, Tsang S, Williamson B, Stockert E, Pfreundschuh M, Old LJ (1997). A testicular antigen aberrantly expressed in human cancers detected by autologous antibody screening. Proceedings of the National Academy of Sciences of the United States of America.

[29] Scanlan MJ, Gordon CM, Williamson B, Lee SY, Chen YT, Stockert E, Jungbluth A, Ritter G, Jager D, Jager E, Knuth A, Old LJ (2002). Identification of cancer/testis genes by database mining and mRNA expression analysis. International journal of cancer Journal international du cancer.

[30] Yokoe T, Tanaka F, Mimori K, Inoue H, Ohmachi T, Kusunoki M, Mori M (2008). Efficient identification of a novel cancer/testis antigen for immunotherapy using three-step microarray analysis. Cancer research.

[31] Chen YT, Venditti CA, Theiler G, Stevenson BJ, Iseli C, Gure AO, Jongeneel CV, Old LJ, Simpson AJ (2005). Identification of CT46/HORMAD1, an immunogenic cancer/testis antigen encoding a putative meiosis-related protein. Cancer immunity.

[32] Chen YT, Scanlan MJ, Venditti CA, Chua R, Theiler G, Stevenson BJ, Iseli C, Gure AO, Vasicek T, Strausberg RL, Jongeneel CV, Old LJ, Simpson AJ (2005). Identification of cancer/testis-antigen genes by massively parallel signature sequencing. Proceedings of the National Academy of Sciences of the United States of America.

[33] Almeida LG, Sakabe NJ, deOliveira AR, Silva MC, Mundstein AS, Cohen T, Chen YT, Chua R, Gurung S, Gnjatic S, Jungbluth AA, Caballero OL, Bairoch A, Kiesler E, White SL, Simpson AJ (2009). CTdatabase: a knowledge-base of high-throughput and curated data on cancer-testis antigens. Nucleic acids research.

[34] Stevenson BJ, Iseli C, Panji S, Zahn-Zabal M, Hide W, Old LJ, Simpson AJ, Jongeneel CV (2007). Rapid evolution of cancer/testis genes on the X chromosome. BMC Genomics.

[35] Zhang Q, Su B (2014). Evolutionary origin and human-specific expansion of a cancer/testis antigen gene family. Molecular biology and evolution.

[36] Sang M, Wang L, Ding C, Zhou X, Wang B, Wang L, Lian Y, Shan B (2011). Melanoma-associated antigen genes - an update. Cancer letters.

[37] Liu Y, Zhu Q, Zhu N (2008). Recent duplication and positive selection of the GAGE gene family. Genetica.

[38] Gjerstorff MF, Ditzel HJ (2008). An overview of the GAGE cancer/testis antigen family with the inclusion of newly identified members. Tissue antigens.

[39] Killen MW, Taylor TL, Stults DM, Jin W, Wang LL, Moscow JA, Pierce AJ (2011). Configuration and rearrangement of the human GAGE gene clusters. American journal of translational research.

[40] Gjerstorff MF, Ditzel HJ (2012). Limited SP17 expression within tumors diminishes its therapeutic potential. Tissue antigens.

[41] Mamsen LS, Brochner CB, Byskov AG, Mollgard K (2012). The migration and loss of human primordial germ stem cells from the hind gut epithelium towards the gonadal ridge. The International journal of developmental biology.

[42] Old LJ (2007). Cancer is a somatic cell pregnancy. Cancer immunity.

[43] Silva WA, Gnjatic S, Ritter E, Chua R, Cohen T, Hsu M, Jungbluth AA, Altorki NK, Chen YT, Old LJ, Simpson AJ, Caballero OL (2007). PLAC1, a trophoblast-specific cell surface protein, is expressed in a range of human tumors and elicits spontaneous antibody responses. Cancer immunity.

[44] Acevedo HF, Tong JY, Hartsock RJ (1995). Human chorionic gonadotropin-beta subunit gene expression in cultured human fetal and cancer cells of different types and origins. Cancer.

[45] Greve KB, Lindgreen JN, Terp MG, Pedersen CB, Schmidt S, Mollenhauer J, Kristensen SB, Andersen RS, Relster MM, Ditzel HJ, Gjerstorff MF (2015). Ectopic expression of cancer/testis antigen SSX2 induces DNA damage and promotes genomic instability. Mol Oncol.

[46] D'Arcy P, Maruwge W, Wolahan B, Ma L, Brodin B (2014). Oncogenic Functions of the Cancer-Testis Antigen SSX on the Proliferation, Survival, and Signaling Pathways of Cancer Cells. PloS one.

[47] Gjerstorff MF, Relster MM, Greve KB, Moeller JB, Elias D, Lindgreen JN, Schmidt S, Mollenhauer J, Voldborg B, Pedersen CB, Bruckmann NH, Mollegaard NE, Ditzel HJ (2014). SSX2 is a novel DNA-binding protein that antagonizes polycomb group body formation and gene repression. Nucleic Acids Res.

[48] Mills AA (2010). Throwing the cancer switch: reciprocal roles of polycomb and trithorax proteins. Nature reviews Cancer.

[49] Por E, Byun HJ, Lee EJ, Lim JH, Jung SY, Park I, Kim YM, Jeoung DI, Lee H (2010). The cancer/testis antigen CAGE with oncogenic potential stimulates cell proliferation by up-regulating cyclins D1 and E in an AP-1- and E2F-dependent manner. The Journal of biological chemistry.

[50] Kim Y, Park D, Kim H, Choi M, Lee H, Lee YS, Choe J, Kim YM, Jeoung D (2013). miR-200b and cancer/testis antigen CAGE form a feedback loop to regulate the invasion and tumorigenic and angiogenic responses of a cancer cell line to microtubule-targeting drugs. The Journal of biological chemistry.

[51] Atanackovic D, Hildebrandt Y, Jadczak A, Cao Y, Luetkens T, Meyer S, Kobold S, Bartels K, Pabst C, Lajmi N, Gordic M, Stahl T, Zander AR, Bokemeyer C, Kroger N (2010). Cancer-testis antigens MAGE-C1/CT7 and MAGE-A3 promote the survival of multiple myeloma cells. Haematologica.

[52] Weeraratne SD, Amani V, Neiss A, Teider N, Scott DK, Pomeroy SL, Cho YJ (2011). miR-34a confers chemosensitivity through modulation of MAGE-A and p53 in medulloblastoma. Neuro-oncology.

[53] Nardiello T, Jungbluth AA, Mei A, Diliberto M, Huang X, Dabrowski A, Andrade VC, Wasserstrum R, Ely S, Niesvizky R, Pearse R, Coleman M, Jayabalan DS, Bhardwaj N, Old LJ, Chen-Kiang S (2011). MAGE-A inhibits apoptosis in proliferating myeloma cells through repression of Bax and maintenance of survivin. Clinical cancer research : an official journal of the American Association for Cancer Research.

[54] Bhatia N, Xiao TZ, Rosenthal KA, Siddiqui IA, Thiyagarajan S, Smart B, Meng Q, Zuleger CL, Mukhtar H, Kenney SC, Albertini MR, Jack Longley B (2013). MAGE-C2 promotes growth and tumorigenicity of melanoma cells, phosphorylation of KAP1, and DNA damage repair. The Journal of investigative dermatology.

[55] Kasuga C, Nakahara Y, Ueda S, Hawkins C, Taylor MD, Smith CA, Rutka JT (2008). Expression of MAGE and GAGE genes in medulloblastoma and modulation of resistance to chemotherapy. Laboratory investigation. Journal of neurosurgery Pediatrics.

[56] Yang B, O'Herrin S, Wu J, Reagan-Shaw S, Ma Y, Nihal M, Longley BJ (2007). Select cancer testes antigens of the MAGE-A, -B, and -C families are expressed in mast cell lines and promote cell viability *in vitro* and *in vivo*. The Journal of investigative dermatology.

[57] Monte M, Simonatto M, Peche LY, Bublik DR, Gobessi S, Pierotti MA, Rodolfo M, Schneider C (2006). MAGE-A tumor antigens target p53 transactivation function through histone deacetylase recruitment and confer resistance to chemotherapeutic agents. Proceedings of the National Academy of Sciences of the United States of America.

[58] Doyle JM, Gao J, Wang J, Yang M, Potts PR (2010). MAGE-RING protein complexes comprise a family of E3 ubiquitin ligases. Molecular cell.

[59] Yang B, O'Herrin SM, Wu J, Reagan-Shaw S, Ma Y, Bhat KM, Gravekamp C, Setaluri V, Peters N, Hoffmann FM, Peng H, Ivanov AV, Simpson AJ, Longley BJ (2007). MAGE-A, mMage-b, and MAGE-C proteins form complexes with KAP1 and suppress p53-dependent apoptosis in MAGE-positive cell lines. Cancer research.

[60] Marcar L, Maclaine NJ, Hupp TR, Meek DW (2010). Mage-A cancer/testis antigens inhibit p53 function by blocking its interaction with chromatin. Cancer research.

[61] Cilensek ZM, Yehiely F, Kular RK, Deiss LP (2002). A member of the GAGE family of tumor antigens is an anti-apoptotic gene that confers resistance to Fas/CD95/APO-1, Interferon-gamma, taxol and gamma-irradiation. Cancer Biol Ther.

[62] Kular RK, Yehiely F, Kotlo KU, Cilensek ZM, Bedi R, Deiss LP (2009). GAGE, an antiapoptotic protein binds and modulates the expression of nucleophosmin/B23 and interferon regulatory factor 1. Journal of interferon & cytokine research : the official journal of the International Society for Interferon and Cytokine Research.

[63] Zeng Y, He Y, Yang F, Mooney SM, Getzenberg RH, Orban J, Kulkarni P (2011). The cancer/testis antigen prostate-associated gene 4 (PAGE4) is a highly intrinsically disordered protein. The Journal of biological chemistry.

[64] Smith SK, Kipling D (2004). The role of replicative senescence in cancer and human ageing: utility (or otherwise) of murine models. Cytogenetic and genome research.

[65] Peche LY, Scolz M, Ladelfa MF, Monte M, Schneider C (2012). MageA2 restrains cellular senescence by targeting the function of PMLIV/p53 axis at the PML-NBs. Cell death and differentiation.

[66] Kim Y, Park H, Park D, Lee YS, Choe J, Hahn JH, Lee H, Kim YM, Jeoung D (2010). Cancer/testis antigen CAGE exerts negative regulation on p53 expression through HDAC2 and confers resistance to anti-cancer drugs. The Journal of biological chemistry.

[67] Wong PP, Yeoh CC, Ahmad AS, Chelala C, Gillett C, Speirs V, Jones JL, Hurst HC (2014). Identification of MAGEA antigens as causal players in the development of tamoxifen-resistant breast cancer. Oncogene.

[68] Suyama T, Shiraishi T, Zeng Y, Yu W, Parekh N, Vessella RL, Luo J, Getzenberg RH, Kulkarni P (2010). Expression of cancer/testis antigens in prostate cancer is associated with disease progression. The Prostate.

[69] Jungbluth AA, Ely S, DiLiberto M, Niesvizky R, Williamson B, Frosina D, Chen YT, Bhardwaj N, Chen-Kiang S, Old LJ, Cho HJ (2005). The cancer-testis antigens CT7 (MAGE-C1) and MAGE-A3/6 are commonly expressed in multiple myeloma and correlate with plasma-cell proliferation. Blood.

[70] Zendman AJ, van Kraats AA, den Hollander AI, Weidle UH, Ruiter DJ, van Muijen GN (2002). Characterization of XAGE-1b, a short major transcript of cancer/testis-associated gene XAGE-1, induced in melanoma metastasis. International journal of cancer Journal international du cancer.

[71] Barrow C, Browning J, MacGregor D, Davis ID, Sturrock S, Jungbluth AA, Cebon J (2006). Tumor antigen expression in melanoma varies according to antigen and stage. Clinical cancer research : an official journal of the American Association for Cancer Research.

[72] Luftl M, Schuler G, Jungbluth AA (2004). Melanoma or not? Cancer testis antigens may help. The British journal of dermatology.

[73] Yang F, Zhou X, Miao X, Zhang T, Hang X, Tie R, Liu N, Tian F, Wang F, Yuan J (2014). MAGEC2, an epithelial-mesenchymal transition inducer, is associated with breast cancer metastasis. Breast cancer research and treatment.

[74] Shang B, Gao A, Pan Y, Zhang G, Tu J, Zhou Y, Yang P, Cao Z, Wei Q, Ding Y, Zhang J, Zhao Y, Zhou Q (2014). CT45A1 acts as a new proto-oncogene to trigger tumorigenesis and cancer metastasis. Cell death & disease.

[75] Caballero OL, Cohen T, Gurung S, Chua R, Lee P, Chen YT, Jat P, Simpson AJ (2013). Effects of CT-Xp gene knock down in melanoma cell lines. Oncotarget.

[76] De Craene B, Berx G (2013). Regulatory networks defining EMT during cancer initiation and progression. Nature reviews Cancer.

[77] Kim Y, Park H, Jeoung D (2009). CAGE, a cancer/testis antigen, induces c-FLIP(L) and Snail to enhance cell motility and increase resistance to an anti-cancer drug. Biotechnology letters.

[78] Woods K, Pasam A, Jayachandran A, Andrews MC, Cebon J (2014). Effects of epithelial to mesenchymal transition on T cell targeting of melanoma cells. Frontiers in oncology.

[79] Bai Q, Assou S, Haouzi D, Ramirez JM, Monzo C, Becker F, Gerbal-Chaloin S, Hamamah S, De Vos J (2012). Dissecting the first transcriptional divergence during human embryonic development. Stem cell reviews.

[80] Gjerstorff MF, Harkness L, Kassem M, Frandsen U, Nielsen O, Lutterodt M, Mollgard K, Ditzel HJ (2008). Distinct GAGE and MAGE-A expression during early human development indicate specific roles in lineage differentiation. Human reproduction.

[81] Lee EK, Song KA, Chae JH, Kim KM, Kim SH, Kang MS (2015). GAGE12 mediates human gastric carcinoma growth and metastasis. Int J Cancer.

[82] Keeney S, Giroux CN, Kleckner N (1997). Meiosis-specific DNA double-strand breaks are catalyzed by Spo11, a member of a widely conserved protein family. Cell.

[83] Pousette A, Leijonhufvud P, Arver S, Kvist U, Pelttari J, Hoog C (1997). Presence of synaptonemal complex protein 1 transversal filament-like protein in human primary spermatocytes. Human reproduction.

[84] Keeney S (2008). Spo11 and the Formation of DNA Double-Strand Breaks in Meiosis. Genome dynamics and stability.

[85] Erenpreisa J, Cragg MS (2010). MOS, aneuploidy and the ploidy cycle of cancer cells. Oncogene.

[86] Ianzini F, Kosmacek EA, Nelson ES, Napoli E, Erenpreisa J, Kalejs M, Mackey MA (2009). Activation of meiosis-specific genes is associated with depolyploidization of human tumor cells following radiation-induced mitotic catastrophe. Cancer research.

[87] Kalejs M, Ivanov A, Plakhins G, Cragg MS, Emzinsh D, Illidge TM, Erenpreisa J (2006). Upregulation of meiosis-specific genes in lymphoma cell lines following genotoxic insult and induction of mitotic catastrophe. BMC cancer.

[88] Cappell KM, Sinnott R, Taus P, Maxfield K, Scarbrough M, Whitehurst AW (2012). Multiple cancer testis antigens function to support tumor cell mitotic fidelity. Molecular and cellular biology.

[89] Whitehurst AW, Xie Y, Purinton SC, Cappell KM, Swanik JT, Larson B, Girard L, Schorge JO, White MA (2010). Tumor antigen acrosin binding protein normalizes mitotic spindle function to promote cancer cell proliferation. Cancer research.

[90] Gjerstorff MF, Burns J, Ditzel HJ (2010). Cancer-germline antigen vaccines and epigenetic enhancers: future strategies for cancer treatment. Expert opinion on biological therapy.

[91] Odunsi K, Matsuzaki J, Karbach J, Neumann A, Mhawech-Fauceglia P, Miller A, Beck A, Morrison CD, Ritter G, Godoy H, Lele S, duPont N, Edwards R, Shrikant P, Old LJ, Gnjatic S (2012). Efficacy of vaccination with recombinant vaccinia and fowlpox vectors expressing NY-ESO-1 antigen in ovarian cancer and melanoma patients. Proceedings of the National Academy of Sciences of the United States of America.

[92] Jager E, Karbach J, Gnjatic S, Neumann A, Bender A, Valmori D, Ayyoub M, Ritter E, Ritter G, Jager D, Panicali D, Hoffman E, Pan L, Oettgen H, Old LJ, Knuth A (2006). Recombinant vaccinia/fowlpox NY-ESO-1 vaccines induce both humoral and cellular NY-ESO-1-specific immune responses in cancer patients. Proceedings of the National Academy of Sciences of the United States of America.

[93] Dhodapkar MV, Sznol M, Zhao B, Wang D, Carvajal RD, Keohan ML, Chuang E, Sanborn RE, Lutzky J, Powderly J, Kluger H, Tejwani S, Green J, Ramakrishna V, Crocker A, Vitale L (2014). Induction of antigen-specific immunity with a vaccine targeting NY-ESO-1 to the dendritic cell receptor DEC-205. Science translational medicine.

[94] Zhu X, Asa SL, Ezzat S (2008). Fibroblast growth factor 2 and estrogen control the balance of histone 3 modifications targeting MAGE-A3 in pituitary neoplasia. Clinical cancer research : an official journal of the American Association for Cancer Research.

[95] Liu W, Cheng S, Asa SL, Ezzat S (2008). The melanoma-associated antigen A3 mediates fibronectin-controlled cancer progression and metastasis. Cancer research.

[96] Walter S, Weinschenk T, Stenzl A, Zdrojowy R, Pluzanska A, Szczylik C, Staehler M, Brugger W, Dietrich PY, Mendrzyk R, Hilf N, Schoor O, Fritsche J, Mahr A, Maurer D, Vass V (2012). Multipeptide immune response to cancer vaccine IMA901 after single-dose cyclophosphamide associates with longer patient survival. Nature medicine.

[97] Alexandrov LB, Nik-Zainal S, Wedge DC, Aparicio SA, Behjati S, Biankin AV, Bignell GR, Bolli N, Borg A, Borresen-Dale AL, Boyault S, Burkhardt B, Butler AP, Caldas C, Davies HR, Desmedt C (2013). Signatures of mutational processes in human cancer. Nature.

[98] Dudley ME, Yang JC, Sherry R, Hughes MS, Royal R, Kammula U, Robbins PF, Huang J, Citrin DE, Leitman SF, Wunderlich J, Restifo NP, Thomasian A, Downey SG, Smith FO, Klapper J (2008). Adoptive cell therapy for patients with metastatic melanoma: evaluation of intensive myeloablative chemoradiation preparative regimens. Journal of clinical oncology : official journal of the American Society of Clinical Oncology.

[99] Robbins PF, Morgan RA, Feldman SA, Yang JC, Sherry RM, Dudley ME, Wunderlich JR, Nahvi AV, Helman LJ, Mackall CL, Kammula US, Hughes MS, Restifo NP, Raffeld M, Lee CC, Levy CL (2011). Tumor regression in patients with metastatic synovial cell sarcoma and melanoma using genetically engineered lymphocytes reactive with NY-ESO-1. Journal of clinical oncology : official journal of the American Society of Clinical Oncology.

[100] Abate-Daga D, Speiser DE, Chinnasamy N, Zheng Z, Xu H, Feldman SA, Rosenberg SA, Morgan RA (2014). Development of a T cell receptor targeting an HLA-A*0201 restricted epitope from the cancer-testis antigen SSX2 for adoptive immunotherapy of cancer. PloS one.

[101] Hiasa A, Nishikawa H, Hirayama M, Kitano S, Okamoto S, Chono H, Yu SS, Mineno J, Tanaka Y, Minato N, Kato I, Shiku H (2009). Rapid alphabeta TCR-mediated responses in gammadelta T cells transduced with cancer-specific TCR genes. Gene Ther.

[102] Morgan RA, Chinnasamy N, Abate-Daga D, Gros A, Robbins PF, Zheng Z, Dudley ME, Feldman SA, Yang JC, Sherry RM, Phan GQ, Hughes MS, Kammula US, Miller AD, Hessman CJ, Stewart AA (2013). Cancer regression and neurological toxicity following anti-MAGE-A3 TCR gene therapy. Journal of immunotherapy.

[103] Linette GP, Stadtmauer EA, Maus MV, Rapoport AP, Levine BL, Emery L, Litzky L, Bagg A, Carreno BM, Cimino PJ, Binder-Scholl GK, Smethurst DP, Gerry AB, Pumphrey NJ, Bennett AD, Brewer JE (2013). Cardiovascular toxicity and titin cross-reactivity of affinity-enhanced T cells in myeloma and melanoma. Blood.

[104] Cameron BJ, Gerry AB, Dukes J, Harper JV, Kannan V, Bianchi FC, Grand F, Brewer JE, Gupta M, Plesa G, Bossi G, Vuidepot A, Powlesland AS, Legg A, Adams KJ, Bennett AD (2013). Identification of a Titin-derived HLA-A1-presented peptide as a cross-reactive target for engineered MAGE A3-directed T cells. Science translational medicine.

[105] Makin G, Hickman JA (2000). Apoptosis and cancer chemotherapy. Cell and tissue research.

[106] Pennington K, Pulaski H, Pennington M, Liu JR (2010). Too much of a good thing: suicide prevention promotes chemoresistance in ovarian carcinoma. Current cancer drug targets.

[107] Goodyear O, Agathanggelou A, Novitzky-Basso I, Siddique S, McSkeane T, Ryan G, Vyas P, Cavenagh J, Stankovic T, Moss P, Craddock C (2010). Induction of a CD8+ T-cell response to the MAGE cancer testis antigen by combined treatment with azacitidine and sodium valproate in patients with acute myeloid leukemia and myelodysplasia. Blood.

[108] Toor AA, Payne KK, Chung HM, Sabo RT, Hazlett AF, Kmieciak M, Sanford K, Williams DC, Clark WB, Roberts CH, McCarty JM, Manjili MH (2012). Epigenetic induction of adaptive immune response in multiple myeloma: sequential azacitidine and lenalidomide generate cancer testis antigen-specific cellular immunity. British journal of haematology.

[109] Sigalotti L, Coral S, Fratta E, Lamaj E, Danielli R, Di Giacomo AM, Altomonte M, Maio M (2005). Epigenetic modulation of solid tumors as a novel approach for cancer immunotherapy. Semin Oncol.

[110] Issa JP (2003). Decitabine. Current opinion in oncology.

[111] Yan M, Himoudi N, Basu BP, Wallace R, Poon E, Adams S, Hasan F, Xue SA, Wilson N, Dalgleish A, Williams O, Anderson J (2011). Increased PRAME antigen-specific killing of malignant cell lines by low avidity CTL clones, following treatment with 5-Aza-2′-Deoxycytidine. Cancer immunology, immunotherapy : CII.

[112] Cruz CR, Gerdemann U, Leen AM, Shafer JA, Ku S, Tzou B, Horton TM, Sheehan A, Copeland A, Younes A, Rooney CM, Heslop HE, Bollard CM (2011). Improving T-cell therapy for relapsed EBV-negative Hodgkin lymphoma by targeting upregulated MAGE-A4. Clinical cancer research : an official journal of the American Association for Cancer Research.

[113] Hodi FS, O'Day SJ, McDermott DF, Weber RW, Sosman JA, Haanen JB, Gonzalez R, Robert C, Schadendorf D, Hassel JC, Akerley W, van den Eertwegh AJ, Lutzky J, Lorigan P, Vaubel JM, Linette GP (2010). Improved survival with ipilimumab in patients with metastatic melanoma. The New England journal of medicine.

[114] Robert C, Thomas L, Bondarenko I, O'Day S, M DJ, Garbe C, Lebbe C, Baurain JF, Testori A, Grob JJ, Davidson N, Richards J, Maio M, Hauschild A, Miller WH, Gascon P (2011). Ipilimumab plus dacarbazine for previously untreated metastatic melanoma. The New England journal of medicine.

[115] Topalian SL, Sznol M, McDermott DF, Kluger HM, Carvajal RD, Sharfman WH, Brahmer JR, Lawrence DP, Atkins MB, Powderly JD, Leming PD, Lipson EJ, Puzanov I, Smith DC, Taube JM, Wigginton JM (2014). Survival, durable tumor remission, and long-term safety in patients with advanced melanoma receiving nivolumab. Journal of clinical oncology : official journal of the American Society of Clinical Oncology.

[116] Ge Y, Xi H, Ju S, Zhang X (2013). Blockade of PD-1/PD-L1 immune checkpoint during DC vaccination induces potent protective immunity against breast cancer in hu-SCID mice. Cancer letters.

[117] Daftarian P, Song GY, Ali S, Faynsod M, Longmate J, Diamond DJ, Ellenhorn JD (2004). Two distinct pathways of immuno-modulation improve potency of p53 immunization in rejecting established tumors. Cancer research.

[118] Saha A, Chatterjee SK (2010). Combination of CTL-associated antigen-4 blockade and depletion of CD25 regulatory T cells enhance tumour immunity of dendritic cell-based vaccine in a mouse model of colon cancer. Scandinavian journal of immunology.

[119] Pedersen AE, Buus S, Claesson MH (2006). Treatment of transplanted CT26 tumour with dendritic cell vaccine in combination with blockade of vascular endothelial growth factor receptor 2 and CTLA-4. Cancer letters.

