# Supplementary material for: Oncogenic cancer/testis antigens: prime candidates for immunotherapy
Source: Oncotarget. 2015 Jun 30;6(18):15772–87. doi: 10.18632/oncotarget.4694 (PMC4599236; doi:10.18632/oncotarget.4694)
Supplement: Supplementary file 1 [file oncotarget-06-15772-s001.pdf]

Oncogenic cancer/testis antigens: prime candidates for immunotherapy

Supplementary Material

Supplementary Table 1. Chromosome X-encoded cancer/testis antigens. Data obtained from the CTDatase (http://www.cta.lncc.br).

CT antigen gene family	CT antigen identifier	Number of genes	Expression in normal tissues	Germline expression	Immunogenicity
MAGE-A	CT1	14	Testis-selective	Primordial germ cells<br>Spermatogonia<br>Primary spermatocytes	Humoral<br>Cellular
MAGE-B	CT3	6	Testis-selective	NA	Humoral
GAGE	CT4	13-39	Testis-selective	Primordial germ cells<br>Spermatogonia<br>Primary spermatocytes	Humoral<br>Cellular
SSX	CT5	9	Testis-resticted	Spermatogonia<br>Primary spermatocytes	Humoral<br>Cellular
NY-ESO-1	CT6	4	NA	Spermatogonia<br>Primary spermatocytes	Humoral<br>Cellular
MAGE-C1	CT7	2	Testis-restricted	Spermatogonia<br>Primary spermatocytes	Humoral<br>Cellular
MAGE-C2	CT10	1	Testis/brain-restricted	Spermatogonia<br>Primary spermatocytes	Humoral<br>Cellular
SPANX	CT11	12	Testis-restricted	Spermatids<br>Spermatozoa	Humoral<br>Cellular
XAGE	CT12	11	Testis-selective	NA	Humoral<br>Cellular
SAGE1	CT14	1	Testis-restricted	NA	Cellular
PAGE	CT16	7	Testis-restricted<br>Testis/brain-restricted	NA	NA
NA88 (pseudogene)	CT18	1	NA	NA	Cellular
IL13RA	CT19	1	NA	NA	NA
CSAGE	CT24	3	Testis-restricted	NA	Cellular
CAGE	CT26	1	NA	Spermatids	Humoral

				Spermatozoa	
HOM-TES-85	CT28	1	Testis-selective	NA	Humoral
HCA661	CT30	1	Testis-selective	NA	Humoral
NY-SAR-35	CT37	1	Testis-selective	NA	Humoral
FTHL17	CT38	1	Testis-restricted	NA	NA
NXF2	CT39	2	Testis-selective	NA	NA
TAF7L	CT40	1	Testis-selective	NA	NA
FATE	CT43	1	Testis-selective	NA	Humoral
CT45	CT45	6	NA	Spermatogonia Primary spermatocytes	Humoral
CT47	CT47	12	NA	Spermatogonia Primary spermatocytes	NA
CXorf48	CT55	1	Testis-restricted	NA	NA
PASD1	CT63	1	Testis/brain-restricted	NA	Humoral Cellular
CPXCR1	CT77	1	Testis-restricted	NA	NA
Cxorf61	CT83	1	Testis-restricted	NA	NA
PLAC1	CT92	1	Testis-selective	NA	Humoral Cellular
AKAP4	CT99	1	NA	Spermatozoa	Humoral Cellular
PEPP2	CT107	1	NA	NA	NA
FAM46D	CT112	1	NA	NA	Humoral
FAM133A	CT115	1	Testis/brain-restricted	NA	NA
ARX	CT121	1	NA	NA	NA
ZNF645	CT138		NA	Spermatozoa	NA

Supplementary Table 2. Autosomal-encoded cancer/testis antigens. Data obtained from the CTDatase (http://www.cta.lncc.br).

**CT antigen gene family**	**CT antigen identifier**	**Number of genes**	**Expression in normal tissues**	**Germline expression**	**Immunogenicity**
BAGE	CT3	5	Testis-selective	NA	Cellular
SYCP1	CT8	1	Testis-selective	Spermatocytes	Humoral<br>Cellular
BRDT	CT9	1	Testis-selective	NA	NA
HAGE	CT13	1	NA	NA	NA
LIPI	CT17	1	Testis-selective	NA	NA
CTAGE	CT21	3	Testis/brain-selective	NA	Humoral
SPA17	CT22	1	Testis-selective	Spermatozoa	Humoral<br>Cellular
ACRBP	CT23	1	Testis-selective	Spermatozoa	Humoral<br>Cellular
MMA1	CT25	2	NA	NA	NA
BORIS	CT27	1	NA	Spermatogonia<br>Spermatocytes	Humoral
AF15q14	CT29	1	NA	NA	NA
JARID1B	CT31	1	Testis-selective	NA	NA
LDHC	CT32	1	Testis-selective	Spermatozoa	NA
MORC	CT33	1	Testis-selective	NA	NA
SGY-1	CT34	1	NA		
SPO11	CT35	1	Testis-selective	NA	NA
TPX1	CT36	1	NA	NA	NA
TDRD1	CT41	3	Testis-selective	NA	NA
TEX15	CT42	1	Testis-selective	NA	NA
TPTE	CT44	1	Testis-selective	NA	NA
HORMAD	CT46	2	Testis/brain-selective	NA	Humoral
SLCO6A1	CT48	1	Testis-selective	NA	Humoral
TAG	CT49	1	NA	NA	Humoral<br>Cellular
LEMD1	CT50	1	Testis-selective	NA	NA
HSPB9	CT51	1	Testis-selective	Spermatogonia<br>Primary spermatocytes<br>Spermatids	NA
CCDC110	CT52	1	Testis-selective	NA	Humoral<br>Cellular
ZNF165	CT53	1	Testis-selective	NA	Humoral
SPACA3	CT54	1	Testis-selective	Spermatozoa	NA
THEG	CT56	1	Testis-selective	NA	NA
ACTL8	CT57	1	Testis-selective	NA	NA
NLRP4	CT58	1	Testis-selective	NA	NA
COX6B2	CT59	1	Testis-selective	NA	NA
LOC348120	CT60	1	NA	NA	NA
CCDC33	CT61	1	Testis-selective	NA	NA

LOC196993	CT62	1	NA	NA	NA
TULP2	CT65	1	Testis-selective	NA	NA
CT66	CT66	1	Testis-selective	NA	NA
PRSS54	CT67	1	Testis-selective	NA	NA
RBM46	CT68	1	Testis-selective	NA	NA
CT69	CT69	1	Testis-restricted	NA	NA
CT70	CT70	NA	Testis-restricted	NA	NA
SPINLW1	CT71	1	Testis-selective	Spermatozoa	NA
TSSK6	CT72	1	Testis-selective	Spermatozoa	NA
ADAM29	CT73	1	Testis-selective	NA	Humoral
CCDC36	CT74	1	Testis-selective	NA	NA
LOC440934	CT75	1	NA	NA	NA
SYCE1	CT76	1	Testis-selective	Spermatocytes	NA
TSPY1	CT78	7	NA	Spermatogonia	NA
TSGA10	CT79	1	Testis-selective	Spermatozoa	Humoral
PIWIL	CT80	2	Testis-selective	Spermotogonia Spermatocytes	NA
ARMC3	CT81	1	Testis-selective	NA	Humoral
AKAP3	CT82	1	Testis-selective	Spermatozoa	NA
PBK	CT84	1	Testis-selective	NA	NA
C21ORF99	CT85	1	Testis-selective	NA	Humoral
OIP5	CT86	1	Testis-selective	NA	NA
CEP290	CT87	1	Testis-selective	NA	Humoral
CABYR	CT88	1	Testis-selective	Spermatozoa	Humoral
SPAG9	CT89	1	Testis-selective	Spermatozoa	Humoral
MPHOSPH1	CT90	1	Testis-selective	NA	NA
ROPN1	CT91	1	Testis-selective	Spermatozoa	Humoral
CALR3	CT93	1	NA	Spermatids	NA
PRM	CT94	2	NA	Spermatids Spermatozoa	Humoral
CAGE1	CT95	1	NA	NA	NA
CT96	CT96	1	NA	NA	NA
LY6K	CT97	1	NA	NA	Humoral Cellular
IMP-3	CT98	1	NA	NA	Humoral Cellular
DPPA2	CT100	1	NA	Spermatogonia Spermatocytes	Humoral
KIAA0100	CT101	1	NA	NA	Humoral Cellular
DCAF12	CT102	1	NA	Spermatogonia Spermatocytes	Humoral
SEMG1	CT103	1	NA	NA	Humoral
POTE	CT104	7	NA	NA	NA
GOLGAGL2FA	CT105	1	NA	NA	Humoral
NUF2	CT106	1	NA	NA	NA
OTOA	CT108	1	NA	NA	NA
CCDC62	CT109	1	NA	NA	Humoral
GPATCH2	CT110	1	NA	NA	NA

CEP55	CT111	1	NA	NA	Cellular
TEX14	CT113	1	Testis-restricted	Spermatogonia Spermatocytes	NA
CTNNA2	CT114	1	Testis/brain-restricted	NA	NA
LYPD6B	CT116	1	NA	NA	NA
ANKRD45	CT117	1	NA	NA	NA
ELOVL4	CT118	1	NA	NA	NA
IGSF11	CT119	1	NA	NA	Cellular
TMEFF	CT120	2	NA	NA	NA
SPEF2	CT122	1	NA	NA	NA
GPAT2	CT123	1	NA	NA	NA
TMEM108	CT124	1	NA	NA	NA
NOL4	CT125	1	NA	NA	NA
PTPN20A	CT126	1	NA	NA	NA
SPAG4	CT127	1	NA	NA	NA
MAEL	CT128	1	NA	NA	NA
RQCD1	CT129	1	NA	NA	NA
PRAME	CT130	1	NA	NA	Cellular
TEX101	CT131	1	NA	Spermatids Spermatozoa	NA
SPATA19	CT132	1	NA	Spermatozoa	NA
ODF1	CT133	1	NA	NA	NA
ODF2	CT134	1	NA	Spermatogonia Spermatocytes Spermatids	Humoral
ODF3	CT135	1	NA	Spermatocytes	NA
ODF4	CT136	1	NA	Spermatocytes	NA
ATAD2	CT137	1	NA	NA	NA
KIF2C	CT139	1	NA	NA	NA
SPAG1	CT140	1	NA	Spermatozoa	NA
SPAG6	CT141	1	NA	Spermatozoa	Humoral
SPAG8	CT142	1	NA	Spermatozoa	Humoral
SPAG17	CT143	1	NA	Spermatozoa	Humoral
FBXO39	CT144	1	NA	NA	Humoral
RGS22	CT145	1	NA	Spermatogonia Spermatocytes Spermatids	NA
CyclinA1	CT146	1	NA	Spermatocyte	Cellular
KP-OVA52	CT147	1	NA	NA	NA
CCDC83	CT148	1	NA	NA	Humoral
TEKT5	CT149	1	NA	NA	Humoral
NR6A1	CT150	1	NA	NA	NA
TMPRSS12	CT151	1	NA	NA	NA
TPPP2	CT152	1	NA	NA	NA
PRSS55	CT153	1	NA	NA	NA
DMRT1	CT154	1	NA	Spermatogonia	Spermatocytes
HEMGN	CT155	1	NA	NA	NA
DNAJB8	CT156	1	NA	NA	NA
